# Supplementary material for: Methoxyhispolon Methyl Ether, a Hispolon Analog, Thwarts the SRC/STAT3/BCL-2 Axis to Provoke Human Triple-Negative Breast Cancer Cell Apoptosis In Vitro
Source: Biomedicines. 2023 Oct 10;11(10):2742. doi: 10.3390/biomedicines11102742 (PMC10604664; doi:10.3390/biomedicines11102742)
Supplement: Supplementary file 1 [file biomedicines-11-02742-s001.zip › biomedicines-2667054-supplementary.pdf]

Liao, C.-P. et al.

Methoxyhispolon Methyl Ether, A Hispolon Analog, Thwarts the SRC/STAT3/BCL-2 Axis to Provoke Human Triple-Negative Breast Cancer Cell Apoptosis In Vitro

Supplementary Figures

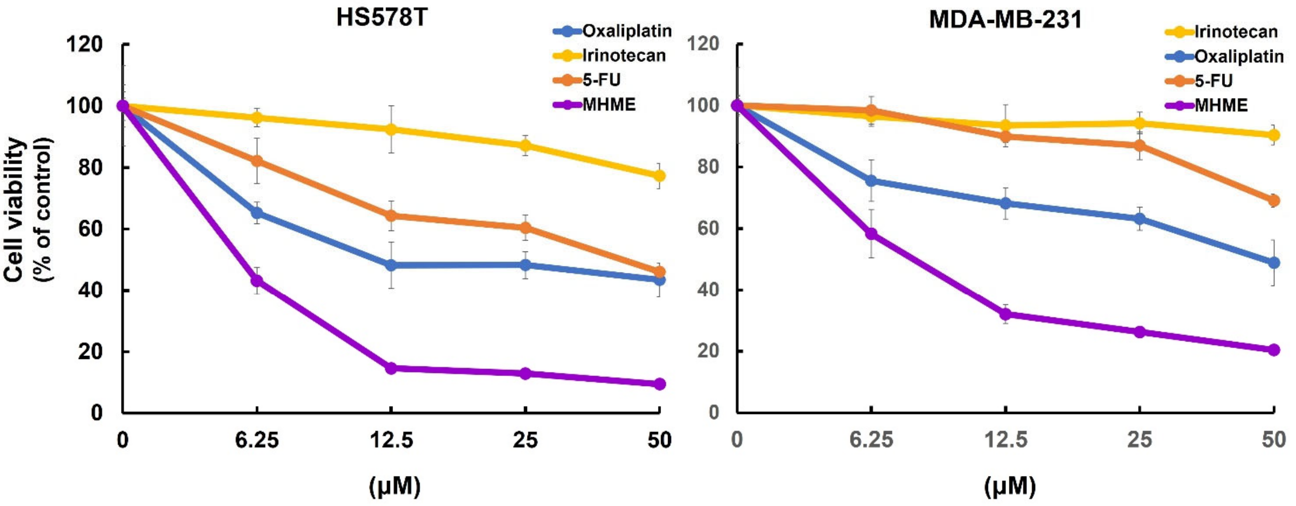

IC_{50} (μM)	Irinotecan	Oxaliplatin	5-FU	MHME
HS578T	> 50	11.84±6.13	43.15±2.93	5.05±0.92

IC_{50} (μM)	Irinotecan	Oxaliplatin	5-FU	MHME
MDA-MB-231	> 50	48.04±3.26	> 50	8.22±0.84

Figure S1. Comparison of the *in vitro* TNBC cytotoxicity of MHME with standard chemotherapeutics. Human TNBC cell lines HS578T and MDA-MB-231 were treated with graded dosages (0, 6.25, 25, 50 μM) of MHME or several common chemotherapeutics, such as 5-Fluorouracil (5-FU), Irinotecan, and Oxaliplatin. After 48 hours, the viability of drug-treated TNBC cells was determined using the CellTiter 96® AQueous One Solution Cell Proliferation Assay (MTS) assay (Promega; Madison, WI, USA) as described in the *Materials and Methods* section.
